# Neurobiology of Cancer: The Role of β-Adrenergic Receptor Signaling in Various Tumor Environments

**DOI:** 10.3390/ijms21217958

**Published:** 2020-10-26

**Authors:** Boris Mravec, Lubica Horvathova, Luba Hunakova

**Affiliations:** 1 Institute of Physiology, Faculty of Medicine, Comenius University, 813 72 Bratislava, Slovakia; 2 Biomedical Research Center, Institute of Experimental Endocrinology, Slovak Academy of Sciences, 814 39 Bratislava, Slovakia; ueenlack@savba.sk; 3 Institute of Microbiology, Faculty of Medicine, Comenius University, 811 08 Bratislava, Slovakia; Luba.Hunakova@fmed.uniba.sk

**Keywords:** β-blockers, cancer, epinephrine, macroenvironment, microenvironment, norepinephrine, psychosocial factors, spirituality, stress, sympathoadrenal system

## Abstract

The development and progression of cancer depends on both tumor micro- and macroenvironments. In addition, psychosocial and spiritual “environments” might also affect cancer. It has been found that the nervous system, via neural and humoral pathways, significantly modulates processes related to cancer at the level of the tumor micro- and macroenvironments. The nervous system also mediates the effects of psychosocial and noetic factors on cancer. Importantly, data accumulated in the last two decades have clearly shown that effects of the nervous system on cancer initiation, progression, and the development of metastases are mediated by the sympathoadrenal system mainly via β-adrenergic receptor signaling. Here, we provide a new complex view of the role of β-adrenergic receptor signaling within the tumor micro- and macroenvironments as well as in mediating the effects of the psychosocial and spiritual environments. In addition, we describe potential preventive and therapeutic implications.

## 1. Introduction

Processes related to the transformation of normal cells to cancerous ones and those related to the proliferation of cancer cells and development of metastases are tightly modulated by both tumor micro- and macroenvironments. Whereas the tumor microenvironment represents a milieu in which transformation occurs and cancer cells interact with cells of the surrounding tissue (e.g., local immune and other stromal cells) and tissue structures (e.g., blood and lymphatic vessels, matrix), the macroenvironment is essential, especially for the systemic immune response of the host to cancer and nourishment of cancer tissue.

For centuries, it was suggested that somatic and psychosocial factors may significantly affect cancer development and progression. However, it is only recently that the neuroendocrine–immune pathways and mechanisms interconnecting psychosocial, somatic factors, and cancer have been elucidated in more detail. The sympathoadrenal system plays a crucial role in these pathways and mechanisms. Plenty of data have shown that effector molecules of the sympathoadrenal system, norepinephrine (NE), and epinephrine (EPI), exert stimulatory effects on cancer development and progression [1,2,3,4,5,6]. This effect is mediated via β-adrenergic receptor signaling, especially the β_2_-adrenergic receptor subtype [6,7,8,9].

The stimulatory effects of EPI and NE on cancer result from the modulation of various biological processes at the level of both tumor micro- and macroenvironments [10]. At the level of the tumor microenvironment, the sympathoadrenal system may affect almost all hallmarks of cancer as defined by Hanahan and Weinberg [11]. Furthermore, the sympathoadrenal system also plays a significant role at the level of the tumor macroenvironment. In addition, it also mediates the effects of psychosocial and noetic factors on cancer. However, a more detailed view integrating the role of the sympathoadrenal system and β-adrenergic receptor signaling in modulating processes within the tumor micro- and macroenvironments, as well as their roles in mediating psychosocial and spiritual influences on cancer initiation, progression, and metastasis is missing in scientific literature.

Therefore, the main aim of this review is to introduce a complex view of the role of β-adrenergic receptor signaling at the level of tumor micro- and macroenvironments. In addition, we describe the role of this signaling in the mediation of psychosocial and spiritual influences on cancer. Potential preventive and therapeutic implications in oncology, based on pharmacological and non-pharmacological approaches that attenuate β-adrenergic receptor signaling are also depicted.

## 2. Norepinephrine and Epinephrine, the Effector Molecules of Sympathoadrenal System

EPI and NE are the principal molecules released by the sympathoadrenal system. Whereas sympathetic nerve endings release NE in tissues innervated by sympathetic nerves, the adrenal medulla represents the main source of circulating EPI and to a lesser extent NE (for a review, see [12]).

EPI and NE bind to adrenergic receptors. Whereas EPI exerts a higher affinity to β-subtypes of adrenergic receptors, NE binds with higher affinity to α-subtypes. The activation of adrenergic receptors activates complex intracellular signaling and effector cascades that affects both the cell’s activity and phenotype [13]. The physiological effects of the EPI and NE include muscle contraction/relaxation, exocytosis from endocrine and exocrine glands, the regulation of metabolic processes, modulation of immune cell activity, regulation of the release of hematopoietic cells from bone marrow, and other biological processes [14]. These effects are important, especially during stressful situations, as the sympathoadrenal system represents a crucial component of the neuroendocrine stress response [12]. However, it is necessary to note that components of the sympathoadrenal system might also be active, to some extent, during quiescent conditions [15]. Therefore, EPI and NE might also affect cells during resting conditions, even if their plasma and tissue levels are much lower when compared to levels found during stress [16].

## 3. Norepinephrine and Epinephrine Effects on Cancer

The effects of EPI and NE on cancer have been documented by many in vitro and in vivo studies that have shown increased cancer cell proliferation in cell cultures exposed to NE or EPI, and cancer incidence and progression in animal models of various cancers as a result of exposure of animals to stressors [1,2,17,18,19,20]. On the other hand, approaches reducing the effects of the sympathoadrenal system, including the chemical destruction of sympathetic nerve endings [21,22,23,24], extirpation of the adrenal medulla [25], or blockade of adrenergic receptors by synthetic antagonists [26,27,28,29], exert inhibitory effects on cancer development and growth.

In the last few years, research has elucidated the cellular and molecular mechanisms responsible for the above-mentioned stimulatory effects of NE and EPI on cancer. At present, it is known that NE and EPI may modulate tumorigenesis, the proliferation of cancer cells, and metastasis formation via multiple downstream molecular pathways at both the tumor micro- and macroenvironment levels [30,31,32,33]. This is possible because of the rich distribution of β-adrenergic receptors, not only in the tumor microenvironment [7], but also on various cell types through the body [14]. As mentioned above, the source of the NE and EPI that may activate tumor-related β-adrenergic signaling [30] are sympathetic nerve endings and the adrenal medulla. Within the tumor microenvironment, the stimulatory effect of NE released locally from sympathetic nerve fibers is more expected [30,34,35], not only due to dense perivascular sympathetic innervation [36], but also sympathetic innervation of the tumor parenchyma itself [24,35,37,38], where NE may directly activate β-adrenergic receptors on both tumor and stromal cells. On the other hand, at the level of the tumor macroenvironment, circulating EPI and NE, as well as NE released locally from sympathetic nerve fibers innervating other organs and tissues, especially lymphatic organs [39], represent an additional pathway by which these catecholamines (CAs; in this paper, the only catecholamines mentioned are NE and EPI, not dopamine) may indirectly regulate cancer biology and the direction of oncologic disease [32,40].

In the following two subchapters, we discuss the possible molecular mechanisms and pathways mediating β-adrenergic influences on cancer progression at both tumor micro- and macroenvironment levels.

### 3.1. Effects of β-Adrenergic Signaling at the Level of Tumor Microenvironment

At the level of the tumor microenvironment, NE and EPI potentiate multiple cellular and molecular processes that contribute to the initiation and progression of cancer [30]. In support of this, elevated NE and EPI levels in the tumor microenvironment will often positively correlate with tumor progression [19].

From the point of view of cancer development and progression, it is important to consider that β-adrenergic signaling affects almost all hallmarks of cancer defined by Hanahan and Weinberg [11]. Interestingly, the source of NE that binds to β-adrenergic receptors in cancer tissue might not originate only in the tumor macroenvironment (e.g., adrenal medulla), but the tumor microenvironment itself may also be responsible for synthesis of NE, as it was demonstrated that many types of tumor tissues are innervated by sympathetic nerve fibers. Importantly, the innervation of tumors was proposed as another emerging hallmark of cancer [41]. Recent findings have demonstrated additional, cancer-specific sources of NE. For example, Mauffrey et al. [42] have shown that neuronal progenitor cells localized in the central nervous system are able to infiltrate prostatic tumor tissue, as well as their metastasis, and that these neurons are able to change their phenotype to an adrenergic one. Moreover, data published by Amit et al. [43] indicate that additional sources of CAs may be due to the reprogramming of sensory neurons innervating cancer tissue to an adrenergic phenotype. Then, these neurons might produce local NE or EPI that are released directly into the tumor microenvironment. That these two above-mentioned mechanisms might provide additional sources of CAs in the tumor microenvironment makes it possible that the catecholaminergic influence on the tumor microenvironment is much more complex than previously expected. However, whether the infiltration of tumor tissue by neurons and the reprogramming of neurons innervating tumor tissue to an adrenergic phenotype represent general, cancer-related phenomena needs further investigation.

#### 3.1.1. β-Adrenergic Signaling Induces Genome Instability and Mutation, and Attenuates DNA Damage Repair Mechanisms

It has been suggested that psychosocial stress and increased adrenergic stimulation may promote cancer induction and progression by compromising the genomic integrity of cells, increasing the frequency of somatic DNA mutations, and sensitizing cells to environmental carcinogens [1,10]. Moreover, dysregulated DNA repair processes may lead to the accumulation of DNA damage and promote aberrant genomic events that lead to malignant transformation and tumor progression [1,44].

Lamboy-Caraballo et al. [1], in their in vitro study, investigated the ability of EPI and NE to induce single- or double-strand DNA damage. They have shown that the exposure of epithelial ovarian cancer cells to either EPI or NE led to increased nuclear γ-H2AX (a marker of double-strand DNA breaks) foci formation in tumor cells. Interestingly, NE treatment caused DNA double-strand breaks but not single-strand breaks. Moreover, the authors proved that pre-treatment with propranolol abrogated NE-induced DNA damage, indicating that the observed effect of NE is mediated by β-adrenergic receptors [1]. Flint et al. [45] studied the molecular effects of short-term in vitro exposure of murine 3T3 cells to EPI and NE with a focus on the effects on DNA damage and repair, cell transformation, and changes in mRNA expression of genes specifically involved in DNA damage signaling pathways. They found that short-term exposure (<30 min) to physiological concentrations of EPI or NE induced at least five-fold increases of DNA damage in treated cells compared to untreated controls. Similar to Lamboy-Caraballo et al. [1], propranolol pre-treatment also eliminated the observed increase in damage. However, the authors found no significant effects of NE or EPI on cell cycle regulation. Finally, targeted gene arrays showed that NE and EPI modulated the transcription of genes directly related to DNA damage signaling pathways, including up-regulation of the DNA damage sensors checkpoint kinase 1 and 2 (Chk1 and Chk2), and the protooncogene cell division cycle 25 A (CDC25A), which is involved in cell cycle delay following DNA damage [45].

Despite several studies dealing with NE and EPI effects on the integrity of DNA, the precise molecular and cellular mechanisms of these compounds’ action on genomic stability remain poorly understood. One possible mechanism was outlined by Hara et al. [46], who suggested that the activation of β_2_-adrenergic receptors by EPI or NE stimulates both Gs–protein kinase A (PKA) and β-arrestin-mediated signaling pathways, triggers DNA damage, and suppresses p53 levels, thus synergistically leading to the accumulation of DNA damage [46].

#### 3.1.2. β-Adrenergic Signaling Potentiates Sustained Proliferative Signaling

Cancer cell proliferation represents a crucial step in cancer development and progression [47]. Several in vitro and in vivo studies have shown that NE and EPI, as well as their agonists, promote cell proliferation in different types of cancer (e.g., gastric, colon, oral squamous, prostate, and pancreatic cancer; as well as melanoma and glioblastoma [48,49,50,51,52,53,54]). However, only a few studies elucidated the possible molecular and cellular mechanisms by which NE and EPI exert their pro-proliferative effects on tumor cells in detail. It seems that they play a significant role in processes leading to the proliferation of tumor cells, especially the intracellular cyclic adenosine monophosphate (cAMP)/PKA signaling pathway that induces the activation of various molecules after stimulation of Gαs-coupled β-adrenergic receptors, including proteases that may affect tumor cell proliferation and differentiation, such as phosphoinositide 3-kinases/protein kinase B (PI3K/Akt), rat sarcoma viral oncogene homolog (Ras)- extracellular signal-regulated protein kinases 1 and 2 (ERK1/2), activator protein 1 (AP-1), signal transducer and activator of transcription 3 (Stat3), nuclear factor-κB (NF-kB), cAMP-response element binding protein (CREB), p38 mitogen-activated protein kinase (P38/MAPK), vascular endothelial growth factor (VEGF), interleukin 6 (IL-6), interleukin 8 (IL-8), and metalloproteases (MMP) [47,48,49,52,54]. Moreover, in melanoma, in addition to β_1_- and β_2_-adrenergic receptors, the involvement of β_3_-adrenergic receptors has also been demonstrated. However, while the role of this subtype of adrenergic receptors has been overlooked so far, data indicate that β_3_-adrenergic receptors are crucial for the modulation of melanoma cell proliferation through nitric oxide signaling [47,55].

However, it has to be noted that the effect of NE and EPI on proliferation-related processes is not universally stimulating in all types of cancers. Especially in breast cancer, the pro- or anti-proliferative effect seems to be dependent on the experimental model of cancer used and which subtype of adrenergic receptor was activated [47,56].

Finally, as described by Calvani et al. [57], other types of stromal cells in the tumor microenvironment also express β-adrenergic receptors, and thus, their activation by NE and EPI may also mediate the observed effects on tumor cell proliferation.

#### 3.1.3. β-Adrenergic Signaling Increases Resistance to Cell Death

Resistance to apoptosis is an important hallmark of cancer. Furthermore, many studies have shown that β-adrenergic antagonists may induce pro-apoptotic signaling pathways. For example, Zhang et al. [58] used BALB/c athymic nude mice injected with human ductal pancreatic adenocarcinoma cells, which were subsequently treated with the β_2_-adrenergic receptor antagonist ICI118,551 or with the β_1_-adrenergic receptor antagonist metoprolol. The authors found that β_2_-adrenergic blockade induced G1/S phase arrest and apoptosis in the injected tumor cells in contrast to the β_1_-adrenergic receptor antagonist metoprolol, which did not. Specifically, β_2_-adrenergic receptor antagonist treatment significantly suppressed the expression of the extracellular signal-regulated kinases, Akt, B-cell lymphoma 2 (Bcl-2), cyclin D1, and cyclin E and induced the activation of caspase-3, caspase-9, and Bax [58]. Similar results were also presented by Chin et al. [59], who used a colorectal cancer model in their study. Likewise, they found that the antagonism of β_2_-adrenergic receptors but not β_1_-adrenergic signaling selectively suppressed cell viability, induced G1-phase cell cycle arrest, caused both intrinsic and extrinsic pathways-mediated apoptosis of specific colorectal cancer cells, and inhibited colorectal cancer xenograft growth in vivo. The authors suggested that the observed effect of β_2_-adrenergic receptor antagonists is mediated via the epidermal growth factor receptor (EFGR)-Akt/ERK1/2 signaling pathway. In the study of Wang et al. [60], the effect of propranolol administered at different concentrations and time intervals on apoptosis of the human liver cancer cell lines HepG2 and HepG2.2.15 was investigated. Propranolol treatment induced apoptosis in cultured liver cancer cells and promoted poly (ADP–ribose) polymerase cleavage, decreased the expression of full-length caspase 3, and induced S-phase arrest in both HepG2 and HepG2.2.15 cell lines [60].

#### 3.1.4. β-Adrenergic Signaling Induces Cell Motility and Trafficking and Activates Invasion and Metastasis

Metastases represent the leading cause of death in oncological patients; therefore, the investigation of metastasis-related processes, such as cancer cell motility, trafficking, and invasion is crucial for the identification of new treatment approaches in oncology. However, the factors that drive the invasion of cancer cells are not yet completely understood. Many studies have suggested that one of the driving factors promoting metastasis and the acceleration of cancer progression is β-adrenergic signaling. However, results obtained from many in vitro and in vivo studies are ambiguous and do not assign a clear pro- or anti-invasive role of β-adrenergic signaling.

Gruet et al. [61] have shown that NE treatment increased invasive capacity in all breast cancer cell lines studied, while protein profiling revealed up-regulation of the pro-metastatic gene Ly6/PLAUR Domain-Containing Protein 3 (LYPD3) in NE-treated MDA-MB-468 breast cancer cells. Moreover, the selective β_2_-adrenergic receptor antagonist ICI-118,551 completely abrogated the enhanced migration and significantly decreased the invasive capacity of MDA-MB-468, BT-549, and MCF-7 cells induced by NE treatment [61]. Contrasting findings were published Bravo-Calderón et al. [62], who investigated the cell migration and invasiveness of two oral squamous cell carcinoma cell lines (SCC-9 and SCC-25) in relation to β_2_-adrenergic receptor signaling. They found that NE treatment induced anti-migratory and anti-invasive effects in both studied cell lines and observed that this effect was dose-dependent. Furthermore, when the β_2_-adrenergic receptor antagonist propranolol was applied before administration of NE, there was an attenuation of the effects of NE [62].

The study of Rivero et al. [63] brought ambiguous results. The authors observed anti-migratory and anti-metastatic effects for both salbutamol (β_2_-agonist) as well as propranolol (non-selective β-blocker) in two human breast cancer cell lines. Treatment with both compounds significantly diminished cell migration, while EPI exerted the opposite effects. Moreover, salbutamol inhibited the invasion of both breast cancer cell lines, enhanced adhesion to the extracellular matrix, and decreased the expression of pro-metastatic genes in MDA-MB-231 cells [63]. Finally, Kim et al. [18] have offered another view on the involvement of β-adrenergic signaling in tumor cell migration and invasion. They have focused on cell deformability, which is an important property of tumor cells that enables their invasion. They have shown that the activation of β-adrenergic signaling by agonists reduces the deformability of stiffer and highly metastatic human breast cancer cells that become more invasive in vitro. Moreover, the authors found that β-adrenergic signaling activation also reduced the deformability of ovarian, prostate, melanoma, and leukemia cells [18].

Zhang et al. [2] observed that EPI and NE significantly accelerated gastric cancer cell proliferation, invasion, and viability in cell culture. The authors also found that the chronic restraint stress-induced increase in plasma levels of CAs promoted the in vivo progression and metastasis of gastric cancer. In this study, CAs increased the metastatic ability of primary gastric cancer cells to migrate to distant tissues by an enhancement of metastasis-related protein expression. Moreover, these effects were reversed by the β-blocker propranolol (a non-specific antagonist of β-adrenergic receptors) as well as ICI118,551 (a specific β_2_-adrenergic antagonist), but the selective β_1_-adrenergic antagonist atenolol had no effect on tumor cell proliferation and invasion both in vitro and in vivo [2].

A study by Le et al. [64] investigated the role of lymph vasculature in chronic stress-induced tumor cell dissemination. They showed that chronic stress rearranged lymphatic networks within and around tumors to provide pathways for tumor cell escape. Moreover, VEGF C derived from tumor cells and COX2 inflammatory signaling from macrophages seems to be required for stress-induced lymphatic remodeling. Finally, the authors also focused on the incidence of lymph node metastasis in a cohort of breast cancer patients and showed that β-blocker use was significantly associated with reduced risk of lymph node metastasis [64].

Chang et al. [65] also studied the contribution of β_2_-adrenergic signaling in the progression of tumor cells to metastasis in vivo. They used RNA interference to generate MDA-MB-231HM breast cancer cells deficient in β_2_-adrenergic receptors. This modification of tumor cells reduced the proportion of cells with a mesenchymal-like morphology and reduced tumor cell invasion in vitro. The opposite approach, the overexpression of β_2_-adrenergic receptors in low metastatic MCF-7 breast cancer cells, induced an invasive phenotype. Finally, the authors also found that the knockdown of β_2_-adrenergic receptor signaling in tumor cells significantly reduced the effect of stress on metastasis in vivo [65].

#### 3.1.5. β-Adrenergic Signaling Induces Vascular Remodeling and Stimulates Angiogenesis

Tumor angiogenesis is one of the major prerequisites for tumor progression, as tumor cells rely on an adequate oxygen and nutrient supply, as well as the removal of waste products [66]. Angiogenesis is regulated by a plethora of pro- and anti-angiogenic molecules such as interleukin (IL)-8, tumor necrosis factor (TNF)-α, vascular endothelial growth factor (VEGF), transforming growth factor (TGF)-α, TGF-β, angiogenin, platelet-derived growth factor (PDGF), and fibroblast growth factor (FGF) [67]. Importantly, it seems that β-adrenergic signaling also contributes to tumor angiogenesis [66,68].

Xia et al. [19] investigated the mechanisms underlying tumor neovascularization in two xenograft models of lung cancer in mice. The authors performed in vivo chemical depletion using the neurotoxin 6-hydroxydopamine, which resulted in an attenuation of tumor neovascularization and inhibition of tumor growth. In addition, they treated tumor cells with NE or EPI, which enhanced the expression of VEGF in vitro. Finally, they showed that the observed NE- and EPI-stimulated pro-angiogenic effects were reversed by the adrenergic receptor antagonist propranolol. Since the study also investigated the mechanisms of NE- and EPI-induced macrophage polarization, the authors have suggested that increased VEGF production and stimulated angiogenesis in tumors are a consequence of an M2-polarized phenotype of tumor-associated macrophages induced by NE and EPI [19].

Hulsurkar et al. [17] hypothesized that β-adrenergic signaling activated by behavioral stress induces tumor angiogenesis through inducing the epigenetic regulator histone deacetylase-2 (HDAC2), which suppresses TSP1 expression. To clarify this hypothesis, the authors used an in vitro approach, as well as a xenograft model of prostate cancer in mice and showed that HDAC2 is a direct target of cAMP response element binding protein (CREB) activated by β-adrenergic signaling, and it is necessary for β-adrenergic signaling to induce angiogenesis. They also demonstrated that upon CREB activation, HDAC2 repressed thrombospondin-1 through epigenetic regulation [17].

Nuevo-Tapioles et al. [69] investigated the effect of inhibiting mitochondrial respiration in cancer cells using the β_1_-blocker nebivolol that hinders oxidative phosphorylation by significantly inhibiting Complex I and ATP synthase activities. This inhibition of ATP synthase is exerted by the overexpression and binding of the ATPase Inhibitory Factor 1 (IF1) to the enzyme. Consequently, nebivolol reduced angiogenesis in colon and breast tumors in vivo by arresting endothelial cell proliferation [69].

The lymphatic system, together with blood vessels, plays a significant role in cancer progression and metastatic spread [70,71]. In cancer, lymphatic vessels modulate antitumor immunity and develop an immunosuppressive environment, which promotes immune tolerance to cancer and facilitates tumor growth and spread. Moreover, the lymphatic system promotes the invasive properties of tumor cells and provides a pathway for tumor cell escape and infiltration into regional lymph nodes [71,72]. Furthermore, the sympathetic nerves affect the functions of lymphatic vessels via adrenergic receptors [73]. Therefore, β-adrenergic signaling may affect lymphatic vessels. Importantly, lymphatic vessels in cancer tissue are more densely innervated by sympathetic nerves and exert a higher contractility than lymphatic vessels in non-cancer tissues. In addition, it has been shown that β_2_-adrenergic expression significantly correlates with tumor lymphatic permeation [9]. Moreover, high levels of β_2_-adrenergic expression were also associated with lymph node metastasis in patients with early stage disease [74].

In addition to the regulation of lymphatic vessel functions, adrenergic signaling also affects the development and morphology of these vessels. The vascular endothelial growth factors VEGF-C and VEGF-D, specific lymphangiogenic factors that mediate signals for lymphatic endothelial cell growth and migration by binding to VEGFR-3 receptors, are a target of adrenergic signaling. Le et al. [64] have shown that VEGF-C derived from tumor cells is required for the stress-related induction of lymphatic remodeling. Moreover, the pharmacological inhibition of β-adrenergic signaling by propranolol blocked the effect of chronic stress on lymphatic remodeling in vivo and reduced lymphatic metastasis in both preclinical cancer models and patients with breast cancer.

#### 3.1.6. β-Adrenergic Signaling Induces Avoidance to Immune Destruction

It is generally accepted that the immune system plays a central role in processes related to cancer development and progression, as documented by several findings [75,76,77,78]. Importantly, it was shown that CD8^+^ T cells primarily express β_2_-adrenergic receptors and that signaling through this receptor may inhibit CD8^+^ T cell effector function. In the study of Qiao et al. [79], the authors demonstrated that the mechanism by which β_2_-adrenergic signaling may suppress the effector activity of immune cells is inhibition of CD8^+^ T cell activation by suppressing the required metabolic reprogramming events that accompany the activation of these cells. The role of CD8^+^ T cells and β-adrenergic signaling in the anti-cancer immune response was investigated by Nissen et al. [80]. They studied the effects of chronically elevated β-adrenergic signaling on lymphoma progression and antitumor immunity in the Eμ-myc mouse model of B-cell lymphoma, as well as the impact on innate and adaptive immune responses to cancer immunotherapy. They found that chronic treatment with the non-selective β-agonist isoprenaline promoted lymphoma development and significantly suppressed the proliferation, interferon gamma (IFNγ) production, and cytolytic killing capacity of antigen-specific CD8^+^ T cells. The authors concluded that this inhibition of CD8^+^ T-cell responses to immune modulating antibodies, including anti-PD-1 and anti-4-1BB, resulted in less effective control of lymphoma [80].

#### 3.1.7. β-Adrenergic Signaling Induces Tumor-Promoting Inflammation

Tumor-promoting inflammation is another hallmark of cancer [11], but the role of adrenergic signaling in processes interconnecting inflammation and cancer is not well understood [81]. However, NE is known as a potent pro-inflammatory factor and its intratumoral levels are increased under chronic stress conditions [81,82]. Therefore, the understanding of the molecular basis of β-adrenergic signaling in tumor-promoting inflammation might offer new therapeutic possibilities to block inflammation with consequent effects on tumor growth and metastasis formation.

Nagaraja et al. [81] examined the mechanisms by which adrenergic signaling may increase the production of pro-inflammatory metabolites in ovarian tumors and promote tumor metastasis. The authors demonstrated that prostaglandin E2 plays a key role in NE- and EPI-induced inflammation. They described a novel mechanism of pro-inflammatory prostaglandins production via the β_2_-adrenergic receptors–NF-κB– prostaglandin-endoperoxide synthase 2 (PTGS2)– prostaglandin E2 (PGE2) axis. In the Skov3-ip1 and HeyA8 orthotopic models of ovarian cancer, they showed that the blockade of adrenergic signaling with the non-selective β-blocker propranolol or the β_2_-adrenergic receptor specific blocker terbutaline decreased levels of PTGS2 in tumor cells. Moreover, silencing PTGS2 in Skov3-ip1 cells in vivo completely abrogated stress-mediated changes in tumor growth and metastasis [81].

The role of prostaglandins in β-adrenergic signaling-mediated inflammation was also demonstrated in a randomized, controlled trial performed by Haldar et al. [83]. The authors tested the effect of combined perioperative treatment using the β-blocker propranolol and the COX2-inhibitor etodolac that were administered for 20 perioperative days, starting 5 days before surgery, in patients with colorectal cancer on biomarkers of tumor metastasis, immunity, and inflammation. After surgery, whole genome messenger RNA profiling and transcriptional control pathway analyses were analyzed in excised tumors focusing on the study of pro-metastatic and inflammatory indicators, including epithelial-to-mesenchymal transition, cancer-promoting transcription factors, and tumor-infiltrating leukocytes. The authors found out that this treatment reduced tumor infiltrating CD14^+^ monocytes and CD19^+^ B cells while increasing tumor infiltrating CD56^+^ natural killer cells. Moreover, results of analyses indicated an effect on core transcription regulatory circuitry (CRC)-related transcription factors, including the GATA, STAT, and EGR families as well as the CREB family that mediates the gene regulatory effect of β-adrenergic and prostaglandin signaling.

#### 3.1.8. β-Adrenergic Signaling Affects Cancer Cell Energetics

Tumor cells are characterized by altered energetics, resulting in the metabolic reprogramming of these cells. An example of this is the increase in glycolysis, which is known as the Warburg effect and occurs even under anaerobic conditions. From the point of view of the effects of NE and EPI, it is important to note that these molecules significantly influence the processes involved in the metabolic reprogramming of cells, specifically by influencing the synthesis of molecules such as p53, HIF-1α, and sirtuin 1 (SIRT1). In addition, NE and EPI regulate glucose metabolism and ensure that adequate glycemia is maintained even during hypoxia at the level of the tumor macroenvironment. This is provided by signaling cascades activated by NE and EPI, which target transcriptional complexes that modulate the expression of genes regulating metabolic processes [84].

### 3.2. Effects of β-Adrenergic Signaling at the Level of the Tumor Macroenvironment

The tumor macroenvironment, which is responsible for the systemic modulation of neoplastic disease, is critical for the development and progression of cancer as a multicellular entity surviving in the host. In addition to local intercellular signaling through the surrounding microenvironment, there is bidirectional systemic communication between cancer cells, non-transformed cells, as well as other components of the tumor microenvironment with various other tissues, organs, and systems of the body. This leads to an increased complexity of interactions between cancer and the organism and provides the necessary ability of cancer to adapt to changes and signals coming from the macroenvironment [85,86]. At the same time, cancer is able to affect its macroenvironment by suppressing the immune system, evolving paraneoplastic syndromes [86], influencing and resetting endogenous circadian rhythms [87,88], reprogramming the host metabolism leading to cachexia [89], along with affecting psychological and other processes. Therefore, similarly to the tumor microenvironment, at the level of the tumor macroenvironment and psychospiritual environment, interactions between the tumor and its environment are also bidirectional.

It is necessary to note that the concept of the tumor macroenvironment is still evolving and should comprise the complexity of reciprocal interactions of a malignant tumor with its host as an entire organism, including its higher regulatory levels such as the brain, which integrates neural communications in the body with the signals from the outside world [90]. In this review, we examined the concept of tumor environments, introducing a more complex view of possible interactions between tumor and the organismal environments, including the psychosociocultural context of personality.

**Figure 1:**
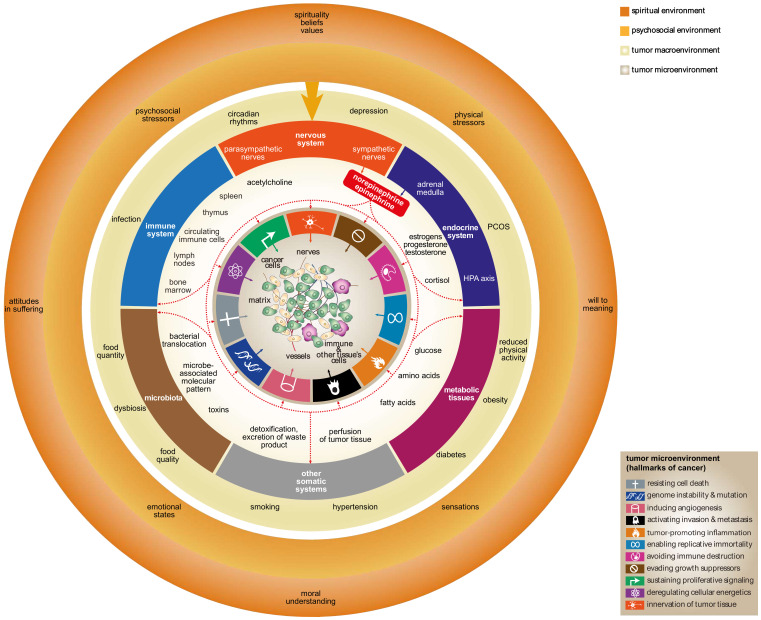
β-adrenergic signaling mediates the effect of the brain on the tumor micro- and macroenvironments (highlighted by red arrows). In addition, β-adrenergic signaling also mediates the effect of psychosocial and noetic environments on cancer development and progression. β-adrenergic signaling affects several components of the tumor macroenvironment, i.e., endocrine, immune, and other somatic systems, including metabolism-related tissues. It affects microbiota as well. Somatic systems, forming the tumor macroenvironment and microbiota, consist of physiological processes, various diseases, and pathological conditions such as circadian rhythms, depression, infection, diabetes, obesity, and hypertension. β-adrenergic signaling directly affects the tumor microenvironment via norepinephrine released by sympathetic nerves innervating tumor tissue and via catecholamines released by the adrenal medulla. Note that in addition to the ten hallmarks of cancer defined by Hanahan and Weinberg [11], tumor innervation was added to the scheme as a new, eleventh hallmark. HPA axis—hypothalamic–pituitary–adrenal axis; PCOS—polycystic ovary syndrome.

There are many tissues and cells in the tumor macroenvironment affecting processes in the tumor microenvironment that are related to cancer development and progression. In our paper, we focus mainly on structures of the tumor macroenvironment, including the nervous and endocrine systems, immune organs and cells, key metabolism-related tissues, and microbiota (Figure 1).

#### 3.2.1. Nervous System

Cancer research has traditionally focused mainly on two areas, alterations in cellular homeostatic regulation (related to DNA mutations and oncogenes) leading to the transformation of a normal cell into a tumor cell and on the role of the immune system in carcinogenesis, cancer progression, and metastasis. Studies, especially from the last two decades, have clearly shown that the nervous system also plays an important role in the development and progression of cancer. These studies use both oncological and neuroscientific approaches, thus creating a basis for the emergence of a new field of cancer research, named “neurobiology of cancer”. The concept of neurobiology of cancer is based on several facts: (a) psychological and environmental factors influence the incidence and progression of cancer; (b) the nervous system modulates antitumor immune responses; (c) cancer tissue is innervated; (d) neurotransmitters affect cancer growth and metastasis; (e) interventions of the nervous system affect the cancer process; (f) cancer tissue affects the activity of the central nervous system (for a review, see [91]).

The neurobiology of cancer provides us with a new view of the mechanisms and pathways that enable psychosocial factors to affect cancer. The nervous system mediates the influence of psychosocial factors on cancer directly via nerves innervating the tumor microenvironment, as well as indirectly, via modulation of the activity of other cells, tissues, and organs (tumor macroenvironment) by nervous and humoral pathways. The indirect effects of the nervous system on cancer play a crucial role in the neural regulation of immune functions. In addition, other components of the tumor macroenvironment are under the influence of the nervous system. The crucial neural pathways mediating the effect of the nervous system on the tumor macroenvironment involve the sympathoadrenal system, which also affects the tumor microenvironment, as mentioned in a previous chapter (Figure 1).

##### The Sympathoadrenal System Affects Metastasis

Adrenergic signaling represents factors playing a fundamental role in metastasis formation as evidenced by the increasing number of studies on this topic. This effect is implemented not only at the level of tumor microenvironment (see Section 3.1.4.), but also at the level of the tumor macroenvironment.

Chen et al. [92] have demonstrated the effect of chronic psychological stress on the lung colonization efficiency of circulating breast cancer cells [93]. The authors found that pre-exposure to chronic stress enhanced the formation of lung metastases and suggested that chronic stress critically influences pre-metastatic lungs before the arrival of disseminated tumor cells through the increased output of monocytes in the pre-metastatic phase and the infiltration of macrophages into the pre-metastatic lung [92].

Recently, the perioperative release of CAs and prostaglandins was shown to facilitate metastasis and reduce disease-free survival in breast cancer patients, while the inhibition of cyclooxygenase 2 (COX-2) and β-adrenergic signaling decreased both epithelial-to-mesenchymal transition and activity of pro-metastatic/pro-inflammatory transcription factors (GATA-1, GATA-2, early-growth-response-3/EGR3, and signal transducer and activator of transcription-3/STAT-3) [27] through suppressing anti-metastatic immunity [94].

In phase II of a randomized controlled trial, Hiller et al. [95] evaluated the effect of a pre-operative β-blockade with propranolol on biomarkers of metastatic potential and the immune cell profile within the primary tumor of patients with breast cancer. They observed that after seven days, pre-operative propranolol treatment down-regulated biomarkers of metastatic potential and inflammation while improving cellular immune response in breast cancer patients [95]. The crucial role of β-adrenergic signaling in metastasis has been covered in a review published by Ricon et al. [96].

#### 3.2.2. Immune System

One of the principal regulators of tumor development, progression and metastasis formation is the immune system. In the context of the tumor environment as defined by Dieterich and Bikfalvi [70], immune system activity is not limited and privileged to a specific part of the tumor environment, but it may affect processes at all its levels [97] from the tumor cell up to the tumor–organismal environment.

The functions of immune organs and cells are modulated by β-adrenergic signaling. At the level of the tumor macroenvironment, the nervous system’s effect on immune function is mediated by NE and EPI, which exert modulatory influence on immune cell activity. These CAs affect circulating immune cells along with primary and secondary lymphatic organs. Importantly, the sympathoadrenal system might also affect immune function via modulation of the development and release of immune cells from bone marrow [98].

It is known that suppression of the antitumor immune response is one of the consequences of chronic stress [32]. Furthermore, the activation of β-adrenergic signaling limits the efficacy of cancer immunotherapy [33]. The main mediators of the immune-related action of CAs are α- and β-adrenergic receptors, which are richly expressed by innate (neutrophils, macrophages, monocytes, mature dendritic cells (DC), and natural killer (NK) cells) as well as adaptive immune cells (T and B cells), with the β_2_-adrenergic receptor as the most highly expressed subtype on both innate and adaptive immune cells [14]. Moreover, T and B cells, as well as hematopoietic stem cells and progenitors, exclusively express this subtype of adrenergic receptor. After their activation by CAs, a cascade of signaling pathways is triggered, resulting in changes in immune cell activity that may also affect their anti-cancer activity. For example, β-adrenergic signaling (a) promotes the polarization of macrophages toward an M2 phenotype and induces changes in cytokine production in favor of anti-inflammatory ones [99], (b) impairs maturation, cytokine production, and antigen presentation of dendritic cells as well as inhibits their migration [32], and (c) suppresses NK cell cytotoxic activity [32]. Moreover, in addition to classical and generally accepted immune mechanisms and mediators of the anti-cancer immune response, there are many studies that highlight the role of other immune factors involved in anti-tumor immunity. For instance, Huang et al. [100] focused on the study of the specific role of granulocytes (polymorphonuclear cells, PMNs) against cancer cells. Specifically, they have suggested that stress promotes cancer incidence by predominantly suppressing the cancer cell killing activity (CKA) of PMNs via one or more stress hormones, namely EPI, NE, and cortisone. The authors showed that the CKA of granulocytes is markedly reduced after stress stimulation in some donors who are psychologically sensitive to stress exposure, with the concentration of plasma stress hormones (EPI, NE, and cortisone) increasing accordingly. Moreover, in vitro co-incubation of PMNs with stress hormones inhibited the CKA of granulocytes. The authors concluded that stress had profound inhibitory effects on the innate anti-cancer functions of PMNs [100]. The innervation of lymphatic organs also plays a fundamental role in the link between the nervous and immune systems [101]. Sympathetic noradrenergic fibers densely innervate primary (thymus and bone marrow) and secondary (lymph nodes and spleen) lymphatic organs and may alter immune responses by releasing neurotransmitters and neuropeptides including NE and its co-transmitter, neuropeptide Y, from sympathetic nerve endings [39,101]. Dysregulation of this interaction may promote the pathogenesis and progression of many diseases, including cancer [101].

#### 3.2.3. Endocrine System

The nervous system modulates the activity and morphology of endocrine glands. Specifically, the hypothalamic–pituitary neuroendocrine axis and sympathetic innervation modulates the release of hormones from endocrine glands. These hormones (e.g., epinephrine, estrogens) might then modulate the development and progression of cancer, especially hormone-dependent types. In addition, sympathetic nerves innervating endocrine grands also exert a trophic effect on these glands. Therefore, the endocrine system is under the complex modulatory influence of the nervous system [102].

#### 3.2.4. Metabolism

β-adrenergic signaling plays a significant role in the modulation of metabolic processes. Crucial metabolic tissues and organs, including the liver, adipose tissue, and skeletal muscles are innervated by sympathetic nerves. Sympathetic innervation modulates both the function and morphology of the above-mentioned components of the metabolic system [103]. Through the modulation of metabolism, the sympathoadrenal system might participate in the regulation of nourishment of cancer tissue.

An altered sympathetic modulation of metabolism might represent a risk factor for cancer development and progression, as it has been suggested that metabolic imbalance significantly affects cancer development and spread [70]. Some metabolic conditions, such as diabetes or obesity, are also considered to be a risk for cancer development [70].

We suggest that cancer itself might affect metabolism via the alteration of sympathetic nerve activity, thereby participating in the development of metabolic dysfunction on a systemic level termed cancer-associated cachexia, which is characterized by the weight loss, skeletal muscle wasting, and atrophy of adipose tissue observed in advanced cancer patients [104]. Importantly, this energy imbalance may be affected by adrenergic signaling. For example, it has been shown that treatment with the specific β_3_-adrenergic antagonist SR59230A (SR) mitigates cancer-associated cachexia through the decreased lipolysis of white adipose tissue, reduces the expression of markers for white adipose tissue browning, and also preserves muscle mass that represents one of the main weapons in the treatment of cancer-associated cachexia [105]. A similar conclusion was published by Salazar-Degracia et al. [106] in a study in which the β_2_-adrenergic agonist formoterol was used. The treatment of cachectic rats with this agonist attenuated structural alterations, atrophy signaling, and molecular perturbations. Namely, in the diaphragm and gastrocnemius of cancer cachectic rats, fiber sizes were reduced. Furthermore, levels of structural alterations, atrophy signaling pathways, proteasome content, protein ubiquitination, autophagy, and myostatin were increased, while those of regenerative and metabolic markers (myoblast determination protein 1 (myoD), mammalian target of rapamycin (mTOR), Akt, and peroxisome proliferator-activated receptor gamma coactivator 1-alpha (PGC-1α)) were decreased.

#### 3.2.5. Microbiota

The microbiota is composed of a vast collection of microorganisms living on the external and internal epithelial surface of the body. Recent findings indicate that an aberrant quality and quantity of microbiota (dysbiosis) is associated with carcinogenesis and cancer progression [107]. These effects are mediated by the induction or promotion of inflammation, modulation of cell growth and proliferation, reduction of immunosurveillance, and alteration of food and drug metabolism or other biochemical functions of the host [107]. Gut bacteria also release toxins that might be responsible for alterations at the level of DNA [108]. In addition, the microbiota is also implicated in cancer cachexia [109].

The largest accumulation of human microbiota is found in the gastrointestinal tract. Importantly, the gut microbiota is subject to sympathoadrenal signaling. It is suggested that CAs can also act on bacteria found in the lumen of the digestive tract. Thus, bacteria are “informed” that a stressor is acting on the body, and during these situations, immune responses may be inhibited, which can be “exploited” by potentially pathogenic forms of the intestinal microbiota [110]. For example, CAs released from sympathetic nerve endings and adrenal medulla may increase the virulence of *Escherichia coli* and *Vibrio parahaemolyticus* enterohemorrhagic strains. This effect might be mediated by the binding of NE and EPI to quorum-sensing *E. coli* regulator C (QseC) molecules, which are a bacterial functional analog of adrenergic receptors [111].

Recently, researchers have started to investigate the microbiota in cancer tissue itself. The term “tumor microbiota” was introduced, describing bacteria present directly in cancer tissue. Importantly, dysbiosis was also found in oropharyngeal, gastric, and colon cancer, as well as in the breast tissue of women with breast cancer, and the pancreatic tissue of patients with pancreatic cancer [112]. Since bacteria activity and morphology might by affected by NE and EPI, it can be hypothesized that β-adrenergic signaling might also play a role in the modulation of tumor microbiota. However, further research will be necessary to elucidate these potential effects.

#### 3.2.6. Somatic Diseases Predisposing to Cancer that Are Characterized by Increased β-Adrenergic Signaling

In addition to physiological conditions, it is necessary to also mention the role of β-adrenergic signaling in cancer during some pathological conditions and somatic diseases. Several pathological conditions, diseases, and behaviors that are also associated with increased cancer incidence (e.g., polycystic ovary syndrome, obesity, hypertension, diabetes, and smoking [113,114,115]) are also associated with increased β-adrenergic signaling [116,117,118,119]. Therefore, an attenuation of β-adrenergic signaling in individuals suffering from the above-mentioned pathological conditions and diseases might represent an additional preventive approach to reducing cancer incidence.

### 3.3. Effects of β-Adrenergic Signaling at the Level of Psychosocial and Spiritual/Noetic Environments

The tumor micro- and macroenvironment are “interconnected” by an immense number of chemical compounds that affect the function and morphology of cancer cell functions. In addition to these two “somatic” environments that are “physically” connected with cancer cells, we also recognize the psychosociospiritual environment, the intangible entity that is able to significantly affect cancer development and progression via the modulation of processes taking part in the brain. The brain is the key component of the tumor macroenvironment that mediates the effect of psychosocial and spiritual environments on cancer.

In cancer patients, besides the psychosocial dimension, the spiritual/noetic dimension may also play a role [120,121], the latter emphasizing the human spiritual needs for meaning [122] shown to be important in promoting well-being in cancer patients [123]. The noetic dimension, dealing with the ontological (ultimate) and the personal (terrestrial) meaning of life [124], expresses the people’s freedom to find their lives meaningful in every situation, choosing their attitude toward the conditions they are not always free to choose [120]. Existential distress (fear of cancer recurrence, death anxiety, demoralization, hopelessness, dignity-related distress, and the desire for hastened death) [125] in cancer patients and survivors requires attention from healthcare professionals searching for appropriate psychosocial interventions [126].

As mechanisms enabling psychosocial factors to affect cancer are being elucidated, psychosomatic medicine also slowly penetrates into standard cancer care [127,128,129]. For example, stress-related circulating CAs were found to be associated with biobehavioral factors and anxiety symptoms in head and neck cancer patients [5]. There is a plethora of psychosocial stressors (e.g., social evaluation, family conflict, rejection/separation, and others), and the interindividual differences in stress responsivity underlying physiological mechanisms of stress-related disorders are large [130]. The inappropriate responsiveness of the stress system to stressors may also account for a number of endocrine, metabolic, immune, and psychiatric alterations, each of which can also contribute to cancer development or progression [131]. A special and powerful source of stress in the context of cancer surgery is also represented by the excess perioperative activation of the sympathetic nervous system, which may significantly facilitate prometastatic processes [96].

## 4. Therapeutic Implications and Future Directions

**Figure 2:**
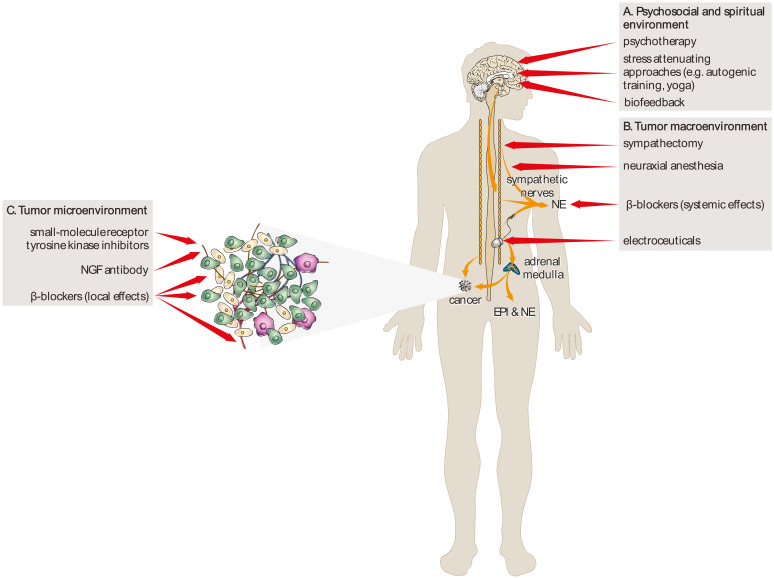
Several pharmacological and non-pharmacological approaches attenuating β-adrenergic signaling might be useful in oncology for the treatment and prevention of cancer. These approaches may act on different levels of the tumor environment, including the psychosocial and spiritual environment (A), tumor macroenvironment (B), and tumor microenvironment. EPI—epinephrine; NE—norepinephrine; NGF—nerve growth factor.

There are many approaches to attenuating β-adrenergic signaling and consequently to inhibit carcinogenesis and cancer growth. These approaches may be focused on different levels of tumor environments (Figure 2).

### 4.1. Pharmacological Approaches

A pharmacological inhibition of neural signaling may become a promising therapeutic target in cancer treatment. β-adrenergic antagonists or blockers are the best studied compounds and have been shown to reduce the risk of, and mortality associated with, multiple types of solid cancer and augment the efficacy of chemotherapeutic agents in preclinical cancer models [38]. The perioperative effects of catecholamine-blocking anesthetics, together with β-adrenergic receptor and prostaglandin inhibition, along with stress-related immunosuppression have shown their immediate therapeutic benefit on cancer outcome in the perioperative setting [132].

β-adrenergic signaling might also be reduced via other pharmacological approaches, including the inhibition of the neurotrophin signaling pathways by nerve growth factor (NGF) antibody tanezumab [133], or by the attenuation of tyrosine kinase receptor A (TrkA) signaling by small-molecule receptor tyrosine kinase inhibitors [134]. In addition, genetic approaches, including manipulation with molecules that determine neuronal fate, such as leukemia inhibitory factor (LIF) and brain-derived neurotrophic factor (BDNF), to push adrenergic nerves toward a cholinergic phenotype [135] may provide additional minimally invasive therapeutic options in oncology. Hiller et al. suggest that neuraxial anesthesia can be used in addition, or as an alternative to general anesthesia, which could help reduce circulating catecholamine levels, inflammation, immunosuppression, and provide an alternative means of achieving sympathetic blockade during cancer surgery [136].

### 4.2. Non-Pharmacological Approaches

These approaches might include tissue-selective surgical sympathectomy [43], psychotherapy [137,138,139], biofeedback [128,140], and electroceuticals, which are a new class of medical devices that modulate the progression of diseases via the modulation of neuronal activity (e.g., vagal nerve stimulation) [141].

### 4.3. The Source of Ambiguity

It was found that the effect of psychotherapy or β-blockers depends on several factors. For example, the efficiency of psychotherapy depends on the type of psychological intervention, type of cancer, and social background. Similar to psychotherapy, the outcomes of clinical studies evaluating the effect of β-blockers on the survival of cancer patients depends on several factors, including the type of β-blocker, dose, frequency of administration, and type of cancer. It is necessary to note that some studies have shown that attenuated β-adrenergic signaling is ineffective or even might potentiate cancer growth (for a review, see [142]). To explain the source of ambiguity, we need to consider that β-blockers attenuate signaling related to only one arm of the neuroendocrine stress response, particularly the sympathoadrenal system. Activity of the second arm of neuroendocrine stress response, the hypothalamic–pituitary–adrenocortical (HPA) axis, is not affected by these drugs. Therefore, glucocorticoids released by the HPA axis, which are known for their stimulatory effect on cancer, might be responsible for ambiguous data related to the effect of β-blockers on the survival of cancer patients.

### 4.4. Future Directions

Even if the knowledge about the role of β-adrenergic signaling in cancer gained over the last two decades increased significantly, many questions would remain unanswered. The main question is whether the stimulatory effect of β-adrenergic signaling on cancer represents general phenomena or if it applies only to certain types of cancers. To answer this question, new prospective clinical studies employing β-blocker treatment in patients with different cancers need to be performed. These studies might also determine the optimal dosage and treatment duration of β-blocker treatment of cancer patients. In addition, it will be necessary to also characterize in detail the role of other subtypes of adrenergic receptors in cancer initiation, progression, and metastasis. Moreover, the potential cancer-related effects of other drugs that affect β-adrenergic signaling, e.g., those antidepressants that modulate noradrenergic neurotransmission, need to be elucidated as well.

## 5. Conclusions

As we described in this review, β-adrenergic signaling represents crucial mechanisms enabling somatic, psychosocial, and noetic factors to affect cancer. The importance of this signaling is supported by preclinical and clinical findings showing that the attenuation of β-adrenergic signaling reduced cancer development and progression both in animal models of cancer as well as in cancer patients. However, data from clinical studies are not completely unequivocal. Therefore, the more precise characterization of factors responsible for this observed ambiguity need to be performed before the wider introduction of compounds inhibiting β-adrenergic signaling such as β-blockers in the daily treatment of cancer patients. Similarly, the use of non-pharmacological interventions that attenuate β-adrenergic signaling requires further detailed and multidisciplinary-oriented research.

In conclusion, we urge further the investigation of brain–cancer interactions mediated via β-adrenergic signaling. Research in this field should be multidisciplinary and may also require the involvement of neurobiological approaches in cancer research.
